# Controversies Surrounding *Clostridium difficile* Infection in Infants and Young Children

**DOI:** 10.3390/children1010040

**Published:** 2014-06-13

**Authors:** Maribeth R. Nicholson, Isaac P. Thomsen, Kathryn M. Edwards

**Affiliations:** 1 Division of Pediatric Gastroenterology, Hepatology, and Nutrition, Vanderbilt University School of Medicine, 2200 Children’s Way, Nashville TN 37232, USA; 2 Division of Pediatric Infectious Disease, Vanderbilt Vaccine Research Program, Vanderbilt University School of Medicine, 2200 Children’s Way, Nashville, TN 37232, USA; E-Mail: isaac.thomsen@vanderbilt.edu; 3 Division of Pediatric Infectious Disease, Vanderbilt Vaccine Research Program, Vanderbilt University School of Medicine, 2200 Children’s Way, Nashville, TN 37232, USA; E-Mail: kathryn.edwards@vanderbilt.edu

**Keywords:** *Clostridium difficile*, colonization, neonates, diarrhea

## Abstract

*Clostridium difficile* is a frequent cause of antibiotic-associated diarrhea in adults and older children. However, as many as 80% of infants can be asymptomatically colonized. The reasons for this have not been well established but are believed to be due to differences in toxin receptors or toxin internalization. Determining which children who test positive for *C. difficile* warrant treatment is exceedingly difficult, especially in the setting of increased rates of detection and the rising risk of disease in children lacking classic risk factors for *C. difficile*.

## 1. Increasing Rates of *Clostridium difficile* Infection

*Clostridium difficile*, a spore forming Gram-positive bacillus, is the most frequent cause of antibiotic associated diarrhea in children and adults. *C. difficile* produces a toxin-mediated intestinal disease with clinical manifestations ranging from asymptomatic colonization to fulminant disease characterized by toxic megacolon, bowel perforation, sepsis, and rarely, death [1]. The development of *C. difficile* infection (CDI) requires alterations in the host intestinal microbiome, most commonly by antibiotics, which allow *C. difficile* to expand and cause mucosal injury and diarrhea. 

*C. difficile* infections have been extensively studied in adults, where both the incidence and severity of CDI are increasing in the United States [2]. Preliminary studies in children also suggest that pediatric disease is on the rise. Zilberberg *et al* reported that the annual rate of pediatric hospitalization with CDI in the USA climbed from 7.24 to 12.8/10,000 hospitalizations during the period from 1997 to 2006 [3]. Another multicenter study from 22 pediatric hospitals across the United States found that the annual incidence of *C difficile*-associated disease in hospitalized children nearly doubled over a five year period (from 26 to 40 per 10,000 admissions from 2001 to 2006) [4]. 

CDI is also developing as a worldwide pathogen. Although a well-established pathogen throughout North America and Europe, recent surveillance also suggests that it is emerging in Asia and other parts of the world where the incidence was initially believed to be low [5]. Unfortunately, the epidemiology of CDI in children is relatively unknown due to limited surveillance. In England and Wales, children less than age 2 are excluded from the otherwise mandatory surveillance required with CDI [6]. 

The epidemiology of CDI in children is also changing, with increased rates of infection in those patients without known risk factors such as antibiotic use or healthcare exposures [7]. The emergence of the epidemic strain of toxin-producing *C. difficile* known as North American pulsed field type 1 (NAP1) may be at least partially responsible for these changes [8]. 

## 2. High Rates of Colonization in Infancy

One of the greatest peculiarities regarding CDI involves the high rate of colonization in infants and the low rates of clinical disease. In healthy infants younger than 1 month of age, *C. difficile* has been recovered from an average of 37% of stools, with a range of 25 to 80% of infants harboring *C. difficile* as a harmless commensal [9,10,11]. Between 1 and 6 months of age, the colonization rate decreases to approximately 30%. This rate continues to decline until age one year when the rate is ~10% in healthy infants. At age three years the rate of colonization reaches 3%, similar to carriage rates previously reported in adults [11]. The high rate of colonization in infants appears to be due to the low capacity of the infant gut to suppress growth of *C. difficile* [12].

Despite this frequent asymptomatic carriage in infants, *C. difficile*-associated diarrheal illness before 12 month of age is exceedingly rare. In a US survey of 20,642 *C. difficile* associated deaths from 1999 to 2004, only 17 occurred in the first year of life [13]. Treating infants with diarrhea with antibiotics directed against *C. difficile* usually does not alter the course of the diarrhea, even if *C difficile* is present in the stool [11]. There also appears to be no correlation between the presence of diarrhea and positive testing for *C. difficile* in infants, supporting the lack of disease in these patients. In two studies of inpatients age 2 years and younger, no significant difference was observed in *C. difficile* positivity between patients with diarrhea and asymptomatic controls [14,15]. With such high rates of colonization in young children, treating all young children with a positive test is not justified. 

Colonization patterns do not appear to vary in infants born by vaginal or cesarean delivery or those with prior administration of antibiotics [16,17]. However, a longer duration of hospital stay does increase the prevalence of the carrier state, and those infants hospitalized in intensive care units *versus* regular nurseries have a higher rate of recovery of *Clostridium difficile* [10]. *C. difficile* spores are notoriously resistant to heat, acid, and antibiotics, suggesting that environmental exposures through hospital settings are an important method of establishing colonization. Also, breast-fed infants are reported to have lower rates of *C. difficile* colonization compared with formula-fed infants but these differences vanish after 12 months [9,18]. Prior studies demonstrated peak colonization around 6 months of age followed by a steady decline [19]. However, a more recent study has demonstrated that colonization may be becoming prolonged, with a steady increase in *C. difficile* in asymptomatic Swedish infants up to 12 months of age [20]. 

## 3. Toxigenic Strains in Infants

The factors that render the neonatal intestine remarkably insensitive to *C. difficile* disease remain unclear. In adults and older children, *C. difficile* causes colitis through the effects of its two toxins, toxin A (TcdA) and toxin B (TcdB), binding to receptors on colonocytes. Receptor-mediated endocytosis is an essential feature of toxin activity, although the mechanism of disease is likely complex and not fully understood. It was initially hypothesized that infants were colonized with non-toxigenic strains of *C. difficile,* and this was the reason for the absence of disease. However, multiple studies have now demonstrated the presence of toxin-producing strains in asymptomatic neonates [21,22]. Recently, a 2008 study of 42 healthy infants colonized with *C. difficile*, found that most strains (71%) were toxin producers and 51% belonged to the 001 or 013 ribotypes which are often associated with symptomatic disease in adults [12]. Interestingly, there have also been reports of CDI in peripartum women whose infants were carriers of the same strains, suggesting that neonates, although themselves asymptomatic, may serve as vectors for *C. difficile* disease in older children and adults [23]. 

The difference between colonization and disease also does not appear to be related to bacterial burden. Adult patients with pseudomembranous colitis harbor *C. difficile* ranging from 10^5^ to 10^9^ bacteria per gram of feces whereas healthy infants have counts as high as 10^8^ bacteria per gram of feces without any evidence of diarrheal disease [11,21]. 

Thus, the mechanism by which infants continue to be resistant to disease in the face of a high burden of toxigenic strains remains a mystery. Some hypotheses suggest that infants may lack the cellular machinery necessary for internalization of the toxin, or that they lack a significant amount of toxin A receptors on the intestinal epithelium. Much of the difficulty in answering this question revolves around the challenge of identifying specific *C. difficile* toxin receptors in humans. A number of receptors for TcdA have been proposed, including α-galactosyl, Lewis X (CD15), Lewis Y, Lewis I, and sucrose isomaltase [24,25,26]. Of these putative TcdA receptors, the most extensively studied is α-galactosyl. A 1987 study in rabbits identified the binding of toxin A to carbohydrates containing α-galactosyl [27]. A follow-up study in humans, however, found that this carbohydrate was not present on normal human cells [25]. Unlike humans, pigs are known to be susceptible to *C. difficile* as neonates. A 2007 study hypothesized that perhaps it was the increased expression of α-galactosyl in pigs that made them susceptible as neonates. However, immunohistochemical studies indicated that the distribution of this carbohydrate on intestinal cell surfaces was much different than that of TcdA binding, suggesting that there was an alternative receptor, at least in pigs, that was critical for toxin binding and has not yet been identified [24]. 

The identification of toxin receptors in humans continues to be elusive. In response to this, investigators have attempted to identify other markers that can predict colonization *versus* pathologic disease. *In vitro*, *C. difficile* toxins activate the p38 pathway and induce multiple pro-inflammatory cytokines [28]. Fecal cytokines including IL-8, lactoferrin, and CXCL-5 have been analyzed in pediatric patients, to attempt to differentiate among *C. difficile* disease states. Although these cytokines were found in larger quantities in children with symptomatic CDI than asymptomatic controls, they failed to differentiate children with CDI from those with other causes of diarrhea [29]. As current recommendations advise testing for *C. difficile* only in those patients with diarrhea, the utility of using cytokines to differentiate between those who are symptomatic from *C. difficile versus* colonized with *C. difficile* and symptomatic from another cause, has not been established. 

## 4. Testing Recommendations

In 2010, the Society of Healthcare Epidemiology of America (SHEA) and the Infectious Diseases Society of America (IDSA) released clinical practice guidelines for CDI in Adults [30]. These guidelines do not address any of the concerns related to disease in children. The American Academy of Pediatrics (AAP) Committee on Infectious Diseases subsequently released a policy statement to provide pediatricians with updated information and recommendations regarding CDI in children. In this statement, the Committee recommended avoiding routine testing for *C. difficile* in children younger than 1 year of age based on the known high rates of colonization and infrequent disease. They also recommended that testing should be limited in this age group to those with Hirschsprung disease or other severe motility disorders or in an outbreak situation. They suggested seeking an alternative etiology even in those with a positive *C. difficile* test result [8]. 

There continues to be controversy however involving testing in children ages 1–3 years. The AAP recommended that testing for *C. difficile* can be considered in children 1–3 years of age with diarrhea, after other causes of diarrhea have been excluded. A positive test result indicates possible CDI [8]. These recommendations rely on the provider to perform testing on high risk children in a step-wise fashion. However, more patients are presenting without identifiable risk factors, and there are constraints to the provider in waiting to test for *C. difficile* until other studies have returned negative, which make these recommendations challenging to follow. 

Adding to the aforementioned challenges, there remains confusion on the actual burden of disease in young children. A recent study from the Emerging Infections Program at the Centers for Disease Control and Prevention published in March 2014 found that young children, age 1–3 years, had the highest incidence of CDI. They also found that rates of severe disease were similar across age groups, with 5% of children age 1 year, 5% of children age 2–3 years, 9% of children age 4–9 years, and 11% of children age 10–17 years suffering from severe CDI. They concluded that due to similar rates of disease severity, the presence of positive *C. difficile* specimens in patients 1 to 3 years of age likely represents infection, as it does in older children [31], which is notably different than the recommendations provided by the AAP as described above [8]. As the authors note, however, this study is limited by a proportion of patients without documented diarrhea, and therefore potentially asymptomatically colonized. Further, due to the overall low rates of severe disease in all pediatric age groups, the study may be underpowered to identify a significant difference in severe disease between age groups if it did exist. This study highlights the need for further evaluation to delineate the true burden of disease in young children. 

## 5. Burden of Testing and Treatment

Due to the difficulties in identifying those patients with symptomatic *C. difficile* disease, many infants and young children with diarrhea are likely tested and treated for CDI. The costs of testing are sizeable. These patients are then subjected to inappropriate antibiotic use and, if in a hospital setting, contact isolation. Prior studies on patients who are placed on contact isolation due to the presence of methicillin resistant *Staphylococcus aureus* (MRSA) demonstrated that these patients receive fewer bedside visits, as well as a tendency towards longer hospital stays and more preventable complications [32]. Contact isolation also frequently persists throughout the duration of hospitalization. This requires caregivers to gown and glove before entering the room and prevents the patient from leaving the room, except under special circumstances, putting an additional strain on the family. 

Toxicity of antimicrobial therapy directed against *C. difficile* is also of significant concern. While the most commonly used first line agent, metronidazole, is frequently well tolerated, it can be associated with severe allergic reactions as well as central nervous system toxicity [33]. The neurotoxicity associated with metronidazole is related to cumulative exposure, and thus occurs at a higher rate following prolonged therapy, as is often used in cases where repeat *C. difficile* testing remains positive. Newer agents with activity against *C. difficile* have been developed and studied in adults, including nitazoxanide, rifaxamin, and fidaxomicin but experience with their use in children is limited to date [34]. Fecal microbiome transplant is a promising therapy for recurrent *Clostridium difficile* in adults and has recently been shown to be effective in a small cohort of children with nine of the ten patients (90%) having resolution of symptoms [35]. The long term safety of this procedure however, remains uncertain. Therefore, identifying those children that truly warrant treatment is paramount. 

## 6. Conclusions

The epidemiology of CDI is changing in children with increasing rates of infection and fewer patients with traditional risk factors such as antibiotic exposures. Identifying those patients who warrant treatment is becoming increasingly challenging for pediatric providers. Until we are better able to understand the complexity of toxin binding and disease pathogenesis with *C. difficile*, we are unlikely to solve the question of why infants and young children may be asymptomatically colonized by a pathogen that can cause severe disease in others. Ideally, once the molecular machinery of *C. difficile* is better understood, we will be able to identify a marker that can distinguish colonized patients from those suffering true disease. In the meantime, deciphering which young pediatric patients with diarrhea warrant treatment for CDI requires a complex analysis of host risk factors, policy recommendations, and clinical acumen. 
